# Combined inhibition of Ref‐1 and STAT3 leads to synergistic tumour inhibition in multiple cancers using 3D and in vivo tumour co‐culture models

**DOI:** 10.1111/jcmm.16132

**Published:** 2020-12-03

**Authors:** Rachel A. Caston, Fenil Shah, Colton L. Starcher, Randall Wireman, Olivia Babb, Michelle Grimard, Jack McGeown, Lee Armstrong, Yan Tong, Roberto Pili, Joseph Rupert, Teresa A. Zimmers, Adily N. Elmi, Karen E. Pollok, Edward A. Motea, Mark R. Kelley, Melissa L. Fishel

**Affiliations:** ^1^ Department of Pediatrics and Herman B Wells Center for Pediatric Research Indiana University School of Medicine Indianapolis IN USA; ^2^ Department of Biochemistry and Molecular Biology Indiana University School of Medicine Indianapolis IN USA; ^3^ Department of Biostatistics Indiana University School of Medicine Indianapolis IN USA; ^4^ Department of Pharmacology and Toxicology Indiana University School of Medicine Indianapolis IN USA; ^5^ Department of Urology Indiana University School of Medicine Indianapolis IN USA; ^6^ Department of Hematology and Oncology Indiana University School of Medicine Indianapolis IN USA; ^7^ Indiana University Simon Comprehensive Cancer Center Indiana University School of Medicine Indianapolis IN USA; ^8^ Department of Surgery Indiana University School of Medicine Indianapolis IN USA; ^9^ Richard L. Roudebush Veterans Administration Medical Center Indianapolis IN USA

**Keywords:** APE1/Ref‐1, Cancer‐associated fibroblasts, Napabucasin, Pancreatic cancer, Ruxolitinib, STAT3, tumor microenvironment

## Abstract

With a plethora of molecularly targeted agents under investigation in cancer, a clear need exists to understand which pathways can be targeted simultaneously with multiple agents to elicit a maximal killing effect on the tumour. Combination therapy provides the most promise in difficult to treat cancers such as pancreatic. Ref‐1 is a multifunctional protein with a role in redox signalling that activates transcription factors such as NF‐κB, AP‐1, HIF‐1α and STAT3. Formerly, we have demonstrated that dual targeting of Ref‐1 (redox factor‐1) and STAT3 is synergistic and decreases cell viability in pancreatic cancer cells. Data presented here extensively expands upon this work and provides further insights into the relationship of STAT3 and Ref‐1 in multiple cancer types. Using targeted small molecule inhibitors, Ref‐1 redox signalling was blocked along with STAT3 activation, and tumour growth evaluated in the presence and absence of the relevant tumour microenvironment. Our study utilized qPCR, cytotoxicity and in vivo analysis of tumour and cancer‐associated fibroblasts (CAF) response to determine the synergy of Ref‐1 and STAT3 inhibitors. Overall, pancreatic tumours grown in the presence of CAFs were sensitized to the combination of STAT3 and Ref‐1 inhibition in vivo. In vitro bladder and pancreatic cancer demonstrated the most synergistic responses. By disabling both of these important pathways, this combination therapy has the capacity to hinder crosstalk between the tumour and its microenvironment, leading to improved tumour response.

## 1 INTRODUCTION

Reduction oxidation (redox) effector factor 1/ apurinic/apyrimidinic endonuclease 1 (Ref‐1/APE1 or Ref‐1) is a protein with multiple distinct functions. The Ref‐1 redox activity reduces critical cysteine residues on transcription factors, such as NFκ‐B, AP‐1, HIF‐1α, STAT3 leading to transcription factor activation. Ref‐1 endonuclease activity is a major component of the base excision repair (BER) pathway. In addition, Ref‐1 plays a role in regulation of the mRNA pool.^1^, ^2^, ^3^, ^4^, ^5^, ^6^ Increase in cell growth, migration, drug resistance and poorer patient prognosis is observed in tumours expressing increased levels of Ref‐1 making it a prominent target for cancer therapy. Our laboratory has been developing specific inhibitors of the redox activity of Ref‐1 and has used APX3330, as well as second‐generation compounds APX2009 and APX2014, in clinical and preclinical studies in cancer as well as ocular neovascular diseases.^7^, ^8^, ^9^ APX3330 slowed tumour growth preclinically and had limited side effects in a phase I clinical trials (NCT003375086).^3^, ^10^

The redox activity of Ref‐1 diminishes cell growth, causes cell cycle arrest and shrinks pancreatic patient‐derived xenografts (PDX) tumours by blocking the tumour cells’ ability to regulate key transcriptions factors such as STAT3 (signalling transducer and activator of transcription‐3), hypoxia inducible factor‐1α (HIF‐1α) and NFκB.^3^ The redox activity of Ref‐1 reduces the oxidized cysteines in STAT3, resulting in increased binding of STAT3 to DNA.^6^ Although Ref‐1 can regulate STAT3 DNA binding and thus expression of its downstream targets such as survivin, it does not affect overall levels of total or phosphorylated STAT3 protein.^6^, ^11^, ^12^ In addition to being under redox control, STAT3 is regulated by phosphorylation which induces homodimerization and promotes translocation of activated STAT3 to the nucleus and subsequent regulation of downstream target genes.^13^ Many STAT3 target genes have been shown to promote inflammation, immune‐escape, tumour invasion and metastasis by up‐regulating cytokines, such as IL‐6, making STAT3 a target of interest in cancer therapy.^3^, ^14^ Two clinically approved drugs for inhibiting STAT3 include ruxolitinib (Rux) and napabucasin (Napa, BBI‐608). Rux blocks JAK signalling upstream of STAT3 and thus inhibits its phosphorylation and activation, and Napa is a cancer stem cell inhibitor that affects STAT3 activation and has been shown to be a substrate of NAD(P)H:quinone oxidoreductase‐1 (NQO1) and to a lesser extent P450 oxidoreductase (POR).^15^, ^16^, ^17^ Napa also reduces the tumour growth of pancreatic cancer line MIA‐PaCa‐2 in vivo and blocks expression of stemness genes in pancreatic cancer cells.^18^

A tumour engages in crosstalk with its microenvironment (TME) which can be influenced by both Ref‐1 and STAT3 signalling pathways. We previously demonstrated that dual targeting of Ref‐1 and STAT3 is synergistic and decreases cell viability in pancreatic cancer cells.^6^ Here, we extend these studies in multiple cancer cell lines and investigate the effects in pancreatic cancer models in vivo. Associated with the actual tumour epithelial cell is a complex stroma composed of CAFs, immune and endothelial cells, and a rich extracellular matrix (ECM).^19^, ^20^ CAFs play a central role in PDAC progression,^21^ and many studies report that CAFs secrete tumour‐promoting growth factors and cytokines.^22^, ^23^, ^24^, ^25^, ^26^, ^27^, ^28^, ^29^, ^30^ CAFs also synthesize and remodel the ECM in the desmoplastic stroma during the progression of the disease. This study examines the one‐two punch of blocking Ref‐1 redox signalling along with STAT3 activation on tumour growth in the presence and absence of the TME. By disabling multiple key pathways, the combination therapy has the potential to disrupt the crosstalk between the tumour and its microenvironment and lead to improved tumour response as well as broad applicability across multiple tumour types.

## 2 MATERIALS AND METHODS

### 2.1 Cell culture

Bladder cancer xenolines (BLCAb001 (RP‐B‐01) and BLCAb002 (RP‐B‐02)^31^, ^32^), malignant peripheral nerve sheath tumour (MPNST) cell lines (ST88‐14, S462 and NF90‐8), colon cancer lines (HCT‐116 and MC38), low passage patient‐derived human pancreatic cancer cell lines (Pa03C, Pa02C, Panc10.05 and Panc198^33^), mouse pancreatic cancer cell lines (*LSL‐Kras^G12D/+^;LSL‐Trp53^R172H/+^;Elas‐Cre^ER^:* KPC2,^34^ *LSL‐Kras^G12D/+^;LSL‐Trp53^R172H/+^;Pdx‐1‐Cre*: KPC32043, KPC32908^35^ and KPC32908‐IL‐6 knockout (IL‐6KO))^36^ and cancer‐associated fibroblasts (CAF19) were maintained at 37°C in 5% CO_2_ and grown in Dulbecco's Modified Eagle's Medium (DMEM; Invitrogen) with 10% Foetal Bovine Serum (FBS; Atlanta Biologicals). ST88‐14 and S462 were received from Dr Andrew Tee (Cardiff University). MPNST cell line NF90‐8 was received from Dr Verena Staedtke (Johns Hopkins University) and low passage patient‐derived cells from Dr Anirban Maitra (Johns Hopkins University). Primary patient‐derived GBM10 (recurrent) and GBM26 (primary) xenolines have been previously described and the original PDX tumours were a kind gift from Dr Jann Sarkaria (Mayo Clinic, Rochester, MN). Cell line identity was confirmed by DNA fingerprint analysis (IDEXX BioResearch) for species and baseline short‐tandem repeat analysis testing. All cell lines were 100% human, and a 9‐marker short‐tandem repeat analysis is on file. Cells were negative for mycoplasma.^37^, ^38^, ^39^ Human MDA‐MB‐231 NQO1+ and NQO1− breast cancer cells were previously generated by the Boothman lab. Briefly, MDA‐MB‐231 cells contain a 609C>T polymorphism (*2) in NQO1, which makes the protein unstable and thus show no enzyme activity.^40^ Cells were stably transfected with a CMV‐driven NQO1 cDNA or the pcDNA3 vector alone as described.^41^ MDA‐MB‐231 cells were grown in high glucose‐containing RPMI 1640 tissue culture medium containing 5% foetal bovine serum and glutamine (2 mmol/L) at 37°C in a 5% CO_2_, 95% air humidified atmosphere. MIA‐PaCa‐2 NQO1 knockout CRISPR clone from MIA‐PaCa‐2 cell line was generated using U6‐gRNA:hPGK‐puro‐2A‐tBFP (LV04) plasmid (Sigma Sanger Clone ID HS5000019237 and HS5000019238^42^) using the manufacturer's procedure (Sigma‐Aldrich). Non‐targeting sgRNA pLV‐U6g‐PPB (Sigma‐Aldrich) was used as a control. sgRNAs targeting unique locations at the NQO1 locus were designed, cloned and validated by Sanger sequencing. Complete NQO1 knockout clone or NQO1 overexpression were verified by Western blot. All cancer cells were authenticated by STR analysis annually and tested for mycoplasma contamination routinely.

Cell proliferation and viability were measured with Alamar Blue assay as previously described.^43^ Briefly, cancer cell lines were seeded between 1250 and 2000 cells/well depending on their growth rate. Viability was measured 72 hours after treatment. For GBM10 (10 000 cells/well) and GBM26 cells (8000 cells/well), cells were seeded overnight on 96 well plates in DMEM (Gibco) containing 10% FBS and treated the next day with Ref‐1 inhibitors or Napa. After 5 days of incubation, cell growth was determined by methylene blue staining.^44^ Each experiment was conducted in triplicate and repeated at least three times. Final DMSO concentration was ≤0.1%. For isogenic MDA‐MB‐231 and MIA‐PaCa‐2 NQO1+ and NQO1− cell lines, the relative survival assays were based on a long‐term, DNA content assessments after 7 doubling times post‐treatment (relative to vehicle‐treated control) performed in 48‐well dishes. Indicated cell lines were treated with specified agents at various doses for 24 hours.^45^ The relative DNA content (a measure of cell growth – adapted from the method of Labarca and Paigen for each treatment (T) condition was determined by the fluorescence of the DNA dye (Hoescht 33258, Sigma) using a plate reader (Victor X) normalized to the vehicle control (C).^46^ All experiments were performed in at least triplicate and the Welch's *t*‐test (two‐tailed) was performed for statistical analysis between NQO1+ vs NQO1−. The relative survival values at different treatment conditions and doses were graphed to obtain the LD_50_ using GraphPad PRISM 8.4.1.

### 2.2 Inhibitors

Small molecule Ref‐1 inhibitors APX3330, APX2009 and APX2014 (Apexian Pharmaceuticals) were prepared and used as previously described.^7^, ^8^, ^47^ For inhibition of STAT3, two inhibitors were used: napabucasin (SelleckChem) which was dissolved in 100% DMSO and stored as a 20 mmol/L stock at −20°C, and JAK1/2 kinase inhibitor, ruxolitinib (SelleckChem) was dissolved in 100% DMSO and stored as a 50 mmol/L stock at −20°C.

### 2.3 Tumour spheroid 3‐dimensional (3D) assays

PDAC cells were grown in co‐culture as 3‐dimensional tumour spheroids as described previously using cancer‐associated fibroblasts (EGFP‐positive) to mimic stroma and pancreatic cancer cells (TdTomato (red)‐positive). The intensity of the red or green signal from the spheroids over time was quantitated as described in our previous studies.^8^, ^43^, ^48^ Briefly, PDAC and CAF cells (500:2000 cell/well, 1:4 ratio) were seeded in ultralow adherence 96‐well plates (Corning Inc) in media containing 5% FBS and 3% reduced growth factor Matrigel (Corning Inc). Spheroids were fed or treated on days 4, 8 and 12 with red and green fluorescence intensity measured on days 4, 8, 12 and 14 following plating using the Thermo ArrayScan (Thermo Fisher Scientific). Fold change was calculated to assess the effect of drug treatment on spheroid growth and was calculated compared to media control.

### 2.4 Western blot analysis

Western Blots were performed using antibodies for p‐STAT3 and STAT3 (Cell Signaling), Vinculin (Sigma) and Actin (NeoMarkers) according to standard protocols and previous publications.^3^, ^6^, ^48^

### 2.5 Intracellular ROS assays

Pancreatic cancer cell lines were seeded at 16 000 cells/well in 96‐well plates. At 80%‐90% confluency, cells were treated with Ref‐1 redox inhibitors APX3330, APX2009, APX2014 and naphthoquinone negative control RN7‐58^49^ (Apexian Pharmaceuticals), napabucasin, and vehicle control (DMSO) in Opti‐MEM (Gibco) and treated for 2 hours at 37°C, 5% CO_2_. CellROX® Green Reagent (Molecular Probes) was added to the drug media to a final concentration 5 μmol/L and incubated with reagent for 30 minutes. Next, media was removed, and three PBS washes were performed. 3.7% formaldehyde was used to fix the cells for 15 minutes. ROS fluorescence was detected at 485/528 excitation/emission (BioTek Synergy H4). Experiments were done in triplicate, and Student's *t*‐test and one‐way ANOVA in Prism (GraphPad) were used for statistical analysis.

### 2.6 RNA isolation and Real time quantitative PCR (qRT‐PCR) on 3D spheroids

3D spheroids of Pa03C cells were allowed to form for 5 days and then treated with APX2009 (5 µmol/L) and Napa (0.25 µmol/L) on Days 5 and 7. Spheroids were collected 24 hours after treatment (Day 8) and RNA extracted according to manufacturer's protocol (Qiagen). Following reverse transcription of 1 μg of RNA to cDNA (Applied Biosystems), qRT‐PCR was performed in a final volume of 20 μL/well using the SYBR Green PCR kit (Applied Biosystems) on the CFX96 Real time PCR detection system (BioRad). Primers for indicated genes were purchased from OriGene. Cycling conditions for qRT‐PCR were: 1 minute at 95°C; 10 minutes at 95°C; 15 seconds at 95°C; 1 minute at 60°C for 40 cycles. Relative changes in mRNA expression levels were assessed by the 2−ΔΔCT method and changes in target gene expression were normalized to β‐Actin gene.

### 2.7 In vivo studies

NSG (NOD.Cg‐Prkdcscid Il2rgtm1Wjl/SzJ) mice of 6‐8 weeks of age were purchased from breeding colony of the In Vivo Therapeutics Core of the Indiana University Simon Comprehensive Cancer Center. Animals were maintained under pathogen‐free conditions under a 12‐hour light‐dark cycle at 22‐24°C and on Teklad Lab Animal Diet (TD 2014, Harlan Laboratories USA) with ad libitum access to sterile tap water. PDAC cell line, Pa03C and CAF cell line, CAF19 were grown in culture and harvested for subcutaneous implant (2.5 × 10^6^ tumour cells and 5 × 10^6^ CAF cells/mouse) in the flank of NSG mice. Tumours were in log phase growth when treatment was started. Tumour volume for the co‐culture was ~150‐200 mm^3^ and the Pa03C was ~70 mm^3^ when treatment started. Animals were then randomized and treated with 50 mg/kg APX3330 (BID, PO, 4% Cremophor:EtOH), 50 mg/kg ruxolitinib (SID, PO, 4% Cremophor:EtOH), 35 mg/kg gemcitabine (every 3‐4 days, IP, PBS), 50 mg/kg Napa (BID, PO, methylcellulose), or vehicle for 5 days on and 2 days off until the vehicle tumours reached 2000 mm^3^ as indicated in the figure legends. All procedures were approved by the Institutional Animal Care and Use Committee at the IU School of Medicine.

### 2.8 Immunohistochemistry

After euthanasia, tumour tissues were harvested, fixed in 10% neutral buffered formalin (NBF), processed for histological analysis. All tissues were processed through graded alcohols, cleared in xylenes, infiltrated with molten paraffin and then embedded in paraffin blocks. Five‐micron thick sections were cut and mounted on slides for staining.

As previously described, the slides were stained for Ref‐1, Masson's trichrome and vimentin in the Indiana University School of Medicine Research Immunohistochemistry Facility.^8^ Stained slides were scanned with an Aperio CS2 Scanscope to generate whole slide images. Aperio (Leica Biosystems) Image Analysis software was used to identify the pixels positive for the diaminobenzidine (DAB) substrate, by colour and optic density. The per cent positive pixels was then calculated for each annotation and averaged within each tissue type.

### 2.9 Statistics

All the experiments were performed at least three independent times and replicates expressed as Average ± Standard Error (SE). Significance was calculated as per either 2‐way ANOVA or unpaired *t*‐test wherever applicable using GraphPad Prism version 8. For qRT‐PCR in the 3D spheroids, analysis of covariance models (ANCOVA) was used to test the difference in the Ct of each target gene compared with APX2009, Napa, vehicle (DMSO) and combination treatment after normalization by reference gene (Actin) as previously described.^50^ Mixed effect repeated measure regression models with random intercept were used to test tumour growth rate of each treatment (ie the regression slope for a particular treatment) and differences in tumour growth rates between a pair of treatments (ie the difference in regression slopes between two treatments) in the in vivo model.^51^ Tumour weights over time were estimated and compared between treatments from the regression models. A *P*‐value of at least <.05 was considered statistically significant. All statistical analysis was conducted using SAS 9.4 (SAS, Inc, Cary, NC, 2016).

## 3 RESULTS

### 3.1 Combination treatment using either ruxolitinib or napabucasin with Ref‐1 redox inhibitors has additive and synergistic effects on cytotoxicity in multiple cancer cell lines

**Figure 1:**
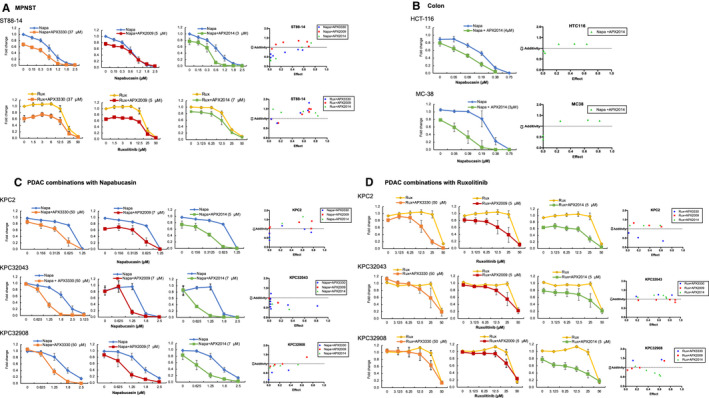
Combination drug treatments with napabucasin or ruxolitinib and Ref‐1 inhibitors in multiple cancer cell lines demonstrate additive to synergistic effects. Cell proliferation following treatment with increasing doses of either napabucasin (in blue) or ruxolitinib (in yellow) in combination with APX inhibitors in MPNST cells ST88‐14 (A), colon HCT‐116 and MC‐38 (B), and pancreatic cancer KPC cell lines (C, D). Combinations with APX3330 are in orange, APX2009 in red, and APX2014 in green. Calcusyn was used to calculate CI values, and those values are graphed to the right of the survival graphs. Cell proliferation was measured by Alamar blue assay and expressed as Fold change compared to Media control (n = 3‐5, avg ± SE)

We investigated the cytotoxic effects of combination treatment with Ref‐1 inhibitors and Rux or Napa using a proliferation‐based assay in multiple human cancer cell lines, including malignant peripheral nerve sheath tumour (MPNST), bladder, colon and glioblastoma, as well as three mouse pancreatic cancer cell lines established from KPC mice (Figure 1, Figure S1). MPNST cell lines ST88‐14, S462 and NF90‐8 had a similar response when challenged with Napa or Rux in combination with Ref‐1 inhibitors APX3330, APX2009 and APX2014, with greater cell death in combinations than with the inhibitors alone (Figure 1A, Figure S1A). MPNST and colon cancer cell lines (MC38 and HCT116, Figure 1B) displayed an increased synergistic effect with Napa and Ref‐1 redox inhibitors at the higher doses of Napa (Isobolograms in Figure 1, Figure S1A, Table S1 and S2). In bladder cancer cell lines B01 and B02, both 2 and 4 μmol/L APX2014 was also more effective in combination with Napa than Napa alone, however the effect in B01 cells was more dramatic and displayed synergy at all doses of Napa (Figure S1B, Isobologram graph in right panel, Table S1 and S2). Importantly, the B01 line has been characterized as inherently resistant to cisplatin, while B02 is sensitive.^12^, ^31^ These data demonstrate that novel combinations can be utilized to treat resistant lines. The recurrent glioblastoma cell line with wt p53, GBM10, demonstrated synergistic effects with combination treatment of APX2009 and Napa while the combination was additive in the primary, p53 mutant GBM26 cells (Figure S1C).

Our previous work demonstrating the efficacy of dual targeting of STAT3 and Ref‐1 was performed in pancreatic cancer cells, MIA‐PaCa‐2, Panc‐1, Panc10.05 and Pa03C using laboratory‐based STAT3 inhibitors, STATTIC and S31‐201.^6^ Here, tumour lines derived from the genetically engineered mouse model KPC also demonstrate promising combination effects, with the addition of Ref‐1 inhibitors causing more cell death than either Rux or Napa alone (Figure 1C,D). Overall, various cancer cell lines consistently responded to combination inhibition of STAT3 signalling and Ref‐1 with additivity or synergy when compared to STAT3 inhibition alone, especially in pancreatic cancer cell lines.

### 3.2 KPC cells that do not express IL‐6 are more sensitive to Ref‐1 inhibitors and combination therapy

**Figure 2:**
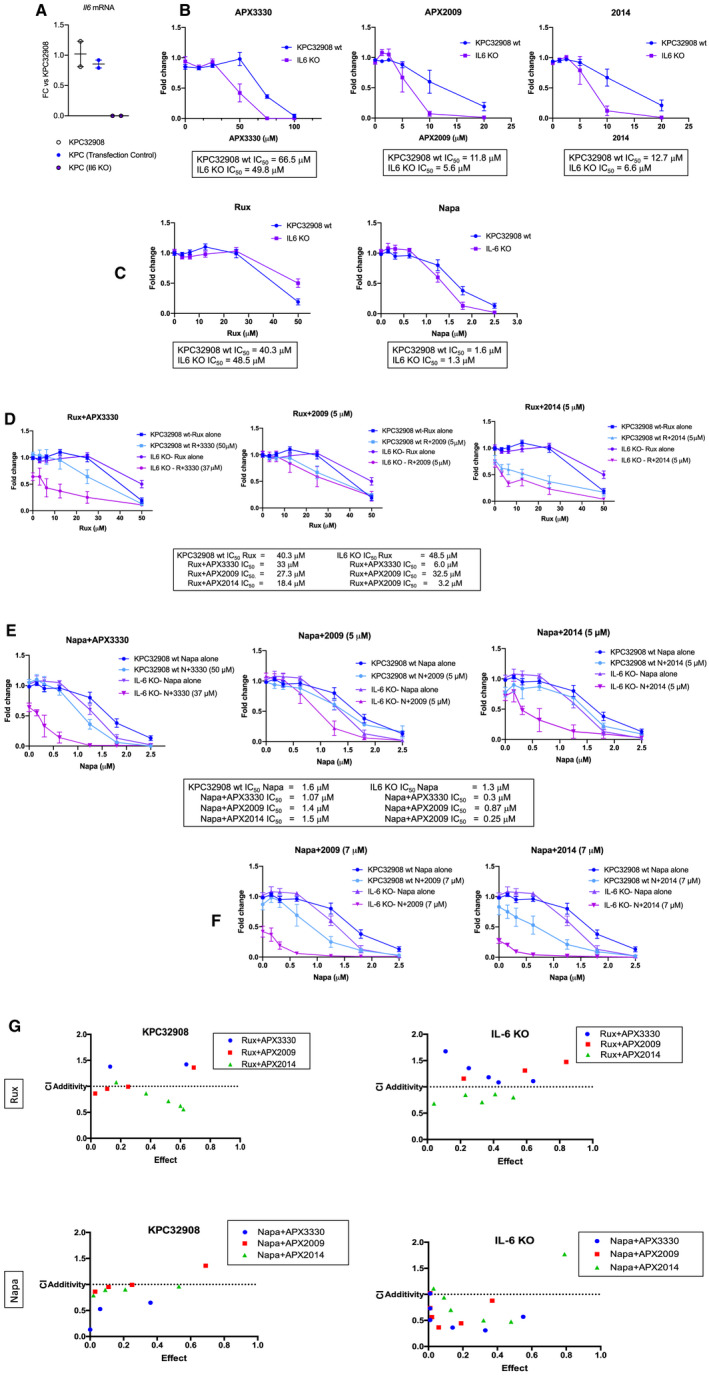
KPC cells that do not express IL‐6 are more sensitive to Ref‐1 inhibition alone and in combination with Napa or Rux. RT‐PCR was used to determine the IL‐6 levels in KPC32908 parent, CRISPR control (blue), and IL‐6 KO (purple) (A, n = 2, avg ± SD). Control cells and IL‐6 KO cells were treated with increasing concentrations of APX compounds (B) as well as Rux and Napa (C). Fold change refers to a comparison of treated to media alone with a DMSO vehicle control included (n = 6‐8, avg ± SE) with IC_50_s listed below. Combination treatment with Rux or Napa with APX compounds are in (D) and (E), respectively. F, the combination of Napa with APX2009 and APX2014 at 7 µmol/L to demonstrate the increased sensitivity of the IL‐6 KO cells. The CI values are graphed in G

The CRISPR‐Cas9 system was used to generate KPC cells that are void of IL‐6 as shown in Figure 2A and as previously described.^36^ The control KPC cells and the IL‐6 knockout (IL‐6 KO) cells were then plated and treated with APX compounds as well as Rux and Napa to determine their sensitivity to these agents. We determined that the slopes were significantly different upon comparison of the KPC32908 cells with the IL‐6 KO cells (Rux and Napa: *P* < .05; APX compounds: *P* ≤ .001). The IC_50_ of APX3330 was decreased by 35% and with APX2009 and 2014 decreased by 50% (Figure 2B). This is further evidence in support of dual targeting of STAT3 and Ref‐1 and the deleterious effects that this combination has on pancreatic cancer cells. The IC_50_s for Rux and Napa were very similar in both KPC wt and IL‐6 KO cells (Figure 2C). Next, combination experiments were performed comparing the effects on cytotoxicity between cells that do not express IL‐6 (IL‐6 KO cells) and the wt cells after the dual targeting strategy. The IL‐6 KO cells were dramatically more sensitive to combination therapy especially with the Napa + APX treatments (Figure 2D‐F). In the IL‐6 KO cells, the doses of APX compounds had to be reduced in order to obtain consistent cytotoxicity data. For example, the concentration of APX3330 in combination with Rux and Napa in the KPC cells was 50 µmol/L, but in the IL‐6 KO cells this was reduced to 37 µmol/L, and even with the lower concentration of Ref‐1 inhibitor the effects on cell survival were greater. Levels of APX3330 in patient serum were 50‐150 µmol/L, well above levels used for these preclinical PDAC studies.^10^ Similar results could be observed with APX2009 and APX2014 (Figure 2F). Isobologram graphs are in Figure 2G demonstrating the synergy that is observed in the IL‐6 KO cells. These cells provide genetic evidence that the inhibition of both STAT3 signalling in concert with Ref‐1 signalling is lethal to pancreatic cancer cells.

### 3.3 Napabucasin and Ref‐1 inhibitors work in concert to prevent PDAC 3D spheroid growth

**Figure 3:**
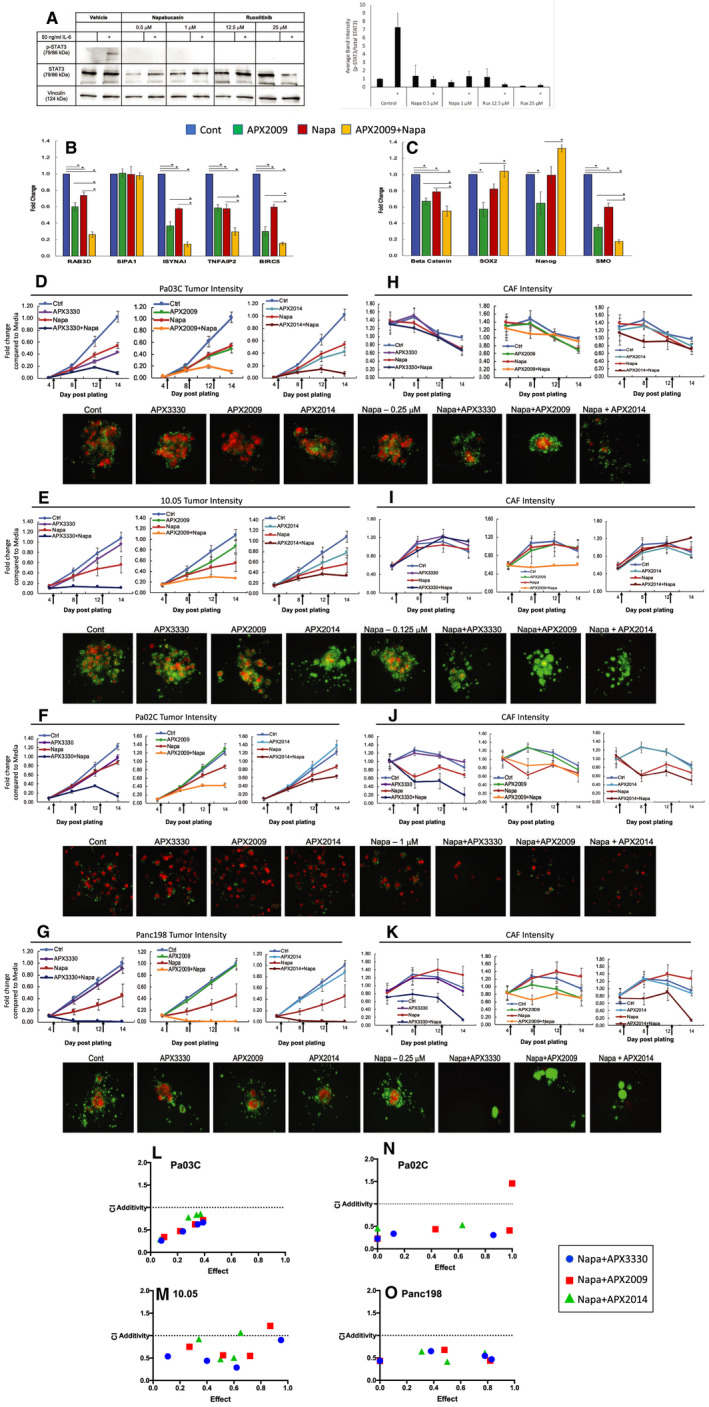
Napabucasin in combination with Ref‐1 inhibition in 3D co‐culture models of pancreatic cancer dramatically effect spheroid growth and signalling. A, Western blot of p‐STAT3 (Y705) and total STAT3 in Pa03C cells treated with Napa or Rux for 4 h and then stimulated with IL‐6 for 15 min at 50 ng/mL (left panel). Quantitation of 3 experiments in right panel. B, C. qPCR evaluation of expression of five gene panel that indicate inhibition of Ref‐1 following treatment (B) or as biomarkers of Napa treatment (C) in 3D spheroid cultures of Pa03C cells (APX2009 – 5 µmol/L, or Napa – 0.25 µmol/L). D‐K, Growth curves of 3D co‐cultures from low passage patient‐derived xenolines [Pa03C (D), Panc10.05 (E), Pa02C (F) and Panc198 (G)]. Intensity of the tumour cells (red, D‐G) as well as the CAFs (green, H‐K) was measured via fluorescence on days 4, 8, 12 and 14 after plating. Representative pictures of the co‐cultures are shown below the graphs. D, 3D Pa03C co‐cultures were treated with Napa (0.125 µmol/L) + APX3330 (35 µmol/L), + APX2009 (5 µmol/L), or + APX2014 (2.5 µmol/L). E, 3D 10.05 co‐cultures were treated with Napa (0.25 µmol/L) + APX3330 (35 µmol/L), + APX2009 (10 µmol/L), or + APX2014 (1 µmol/L). F, 3D Pa02C co‐cultures were treated with Napa (1 µmol/L) + APX3330 (35 µmol/L), + APX2009 (5 µmol/L), or + APX2014 (2.5 µmol/L) and G, Panc198 were treated with Napa (0.25 µmol/L) + APX3330 (35 µmol/L), + APX2009 (10 µmol/L), or + APX2014 (2.5 µmol/L). Fluorescence intensity data was normalized to day 14 media control. Graphs are means with standard error of n = 4‐5. L‐O, Isobolograms to demonstrate the synergy in the tumour cells with the combination of Napa + APX compounds

Napa has previously been reported as a STAT3 inhibitor.^52^ To confirm this activity in PDAC cells, Pa03C cells were incubated for 4 hours with 0.5 or 1 μmol/L Napa, and 12.5 μmol/L or 25 μmol/L Rux as a control for blockade of the phosphorylation of Y705 on STAT3 (Figure 3A). At the end of the treatment, 50 ng/mL IL‐6 was added for 15 minutes, and cells were immediately harvested for western blotting. In the presence of IL‐6, STAT3 was phosphorylated in Pa03C cells as expected. In response to either Napa or Rux, STAT3 phosphorylation was inhibited dramatically (Figure 3A). Expression levels of total STAT3 were unaffected by the treatment. In Pa03C cells, Napa treatment blocks the activity of STAT3 phosphorylation at Y705.

To further investigate the response of PDAC cells to combination treatment as well as APX compounds and Napa, Pa03C cells were grown into 3‐dimensional (3D) tumour spheroids. After five days of growth, spheroid cultures were treated with APX2009 or Napa alone and in combination. Forty‐eight hours later spheroids were treated again, incubated for 24 hours, and then harvested for RNA extraction. qRT‐PCR analysis was performed on genes known to be down‐regulated following Ref‐1 inhibition (RAB3D, SIPA1, ISYNA1, TNFAIP2, BIRC5 [Survivin]; Figure 3B) and markers down‐regulated following Napa treatment (β‐catenin, SOX2, Nanog, SMO; Figure 3C).^16^, ^47^ With the exception of SIPA1, the Ref‐1 responsive genes decreased in expression following treatment with APX2009 (Figure 3B), while SMO and β‐catenin were reduced in expression following Napa treatment (Figure 3C). Combination treatment led to greater down‐regulation than with either APX2009 or Napa alone for the panel of genes tested with the exception of SIPA1, SOX2 and Nanog. From these results, we hypothesize that the combination of STAT3 and Ref‐1 inhibition works synergistically to more dramatically down‐regulate these genes compared to single agent exposure. Additionally, some of these genes appear to be regulated by STAT3 through Ref‐1 redox activity (TNFAIP2, ISYNA1).

Cancer‐associated fibroblasts are an important and complex component of the tumour microenvironment in PDAC.^53^ Therefore, we next explored the effect of CAFs on sensitivity of PDAC tumour cells to combination treatment with Napa and Ref‐1 inhibitors as well as assess the effect of combination treatment on the viability of the CAFs. Previously published data from our lab demonstrated that Rux in combination with APX3330 was synergistic in PDAC cells, but did not affect the CAFs in the same manner.^3^ Here, we evaluate inhibition of STAT3 signalling via Napa in combination with Ref‐1 inhibitors, APX3330, APX2009 and APX2014.^7^, ^12^, ^54^ Napa is able to kill 3D spheroids at much lower doses than ruxolitinib and has been shown to inhibit the growth of xenografted PDAC tumours.^18^ Low passage patient‐derived PDAC cells that express the TdTomato‐red fluorescent protein were grown in 3D co‐culture spheroids with GFP‐labelled CAFs for 14 days (Figure 3D‐K). Pa03C, Panc10.05, Pa02C and Panc198 cells were challenged with Napa in combination with Ref‐1 inhibitors, APX3330 (35 µmol/L), APX2009 (5, 10 µmol/L), or APX2014 (1, 2.5 µmol/L) at 4, 8 and 12 days post‐plating. Spheroid growth was dramatically inhibited by combination treatment of Napa and the Ref‐1 redox inhibitors than by Napa alone in all PDAC lines, with at least a 30% decrease in cell growth between single and combination treatment at concentrations of Ref‐1 inhibitors that can be obtained in vivo (Figure 3D‐G). The co‐cultured CAF cells were less affected by any of the treatment conditions compared to the PDAC cells. In general, combination treatment was much better tolerated in the CAFs compared to the tumours. In the Panc198 spheroids there was some cytotoxicity observed with combination treatment in both the tumours and the CAFs (Figure 3G,K). Synergy evaluation was performed using CalcuSyn, and the isobolograms for tumour cell killing presented in Figure 3L‐O with combination index (CI) values in Tables S1 and S2. Representative pictures of the 3D co‐cultures are shown with TdTomato‐PDAC cells in red and EGFP‐CAFs in green. These results suggest that inhibition of STAT3 and Ref‐1 synergistically and effectively prevents spheroid growth and tumour cell survival with a differential response in the CAFs.

### 3.4 While both napabucasin and APX compounds increase ROS production, mechanisms for ROS production are different

**Figure 4:**
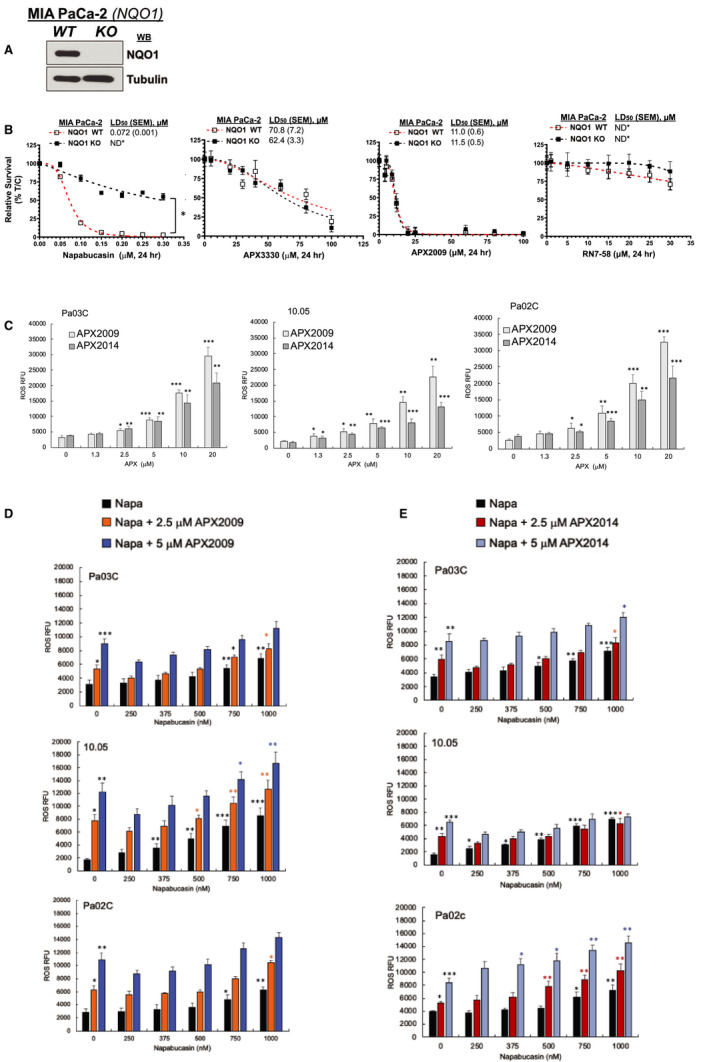
Napabucasin and APX compounds generate ROS in PDAC cells, but only napabucasin is a substrate for NQO1. A, Immunoblotting for NQO1 in MIA‐PaCa‐2 cells. B, Cell survival in NQO1+ and NQO1− cells using relative long‐term survival assays based on DNA content. The Welch's *t*‐test (two‐tailed) was performed for statistical analysis between NQO1+ vs NQO1−, *P < .05 was considered significant. C‐E, CellRox Green was used to quantitate ROS levels after APX and Napa treatment. ROS levels in three pancreatic cancer lines are expressed as Relative fluorescent units (RFU) when treated with either APX2009, APX2014, Napa or the combination for 2 h (Mean ± SE [Unpaired one sided *t*‐test], n = 3, *P < .05, **P < .01 and ***P < .001). Black asterisks are compared to vehicle and the coloured asterisks are compared to the corresponding dose of APX compound

Previous work by Froeling et al established that Napa was bioactivated by NAD(P)H Quinone Oxidoreductase 1 (NQO1), a protein involved in quinone redox cycling,^17^ but the role of NQO1 in bioactivation of APX compounds has not been determined. These studies address our hypothesis that a burst of ROS production may be a part of the mechanism of cell death following combination therapy with Napa and Ref‐1 inhibitors. Using an established model system, paired MIA‐PaCa‐2 and MDA‐MB‐231 cells that were either wild type or NQO1 deficient (Figure 4A, Figure S2A) were treated with increasing concentrations of Napa, APX3330, APX2009, or RN7‐58, the latter an inactive analog of the Ref‐1 inhibitors (Figure 4B, Figure S2B. Only treatment with Napa provided a disparate response, with NQO1 negative cells unaffected by Napa treatment, and NQO1 expressing cells exquisitely sensitive to Napa in both MIA‐PaCa‐2 (Figure 4B) and MDA‐MB‐231 cells (Figure S2B). These data imply that NQO1 is necessary for the activity of Napa, but not required for Ref‐1 inhibitors, APX3330 and APX2009. Moreover, the data also suggest that Ref‐1 inhibitors are not detoxified by NQO1 since NQO1+ and NQO1− isogenic cancer cell lines show closely similar cytotoxicities to Ref‐1 inhibitors.

Investigation of the role of the NQO1 in combination with APX inhibitors and Napa was next investigated in three PDAC cells. To further characterize the role of ROS in cytotoxicity as well as combination treatment, we measured ROS levels in PDAC cells exposed to increasing doses of Ref‐1 inhibitors alone and in combination with Napa (Figure 4D‐F). In Pa03C, 10.05, and Pa02C cells, both Ref‐1 inhibitors generated significant amounts of ROS with APX2009 resulting in ~15% greater levels of ROS than APX2014 at the high dose (20 μmol/L, Figure 4D). In a combination study, Napa and APX2009 or APX2014 gave slightly higher ROS output than Napa alone in all three cell types (Figure 4E,F). The combination of Napa and APX2014 in 10.05 cells did not produce a large increase in ROS levels. Therefore, based on the levels of ROS with combination treatment in three PDAC lines, it is likely that an ROS burst is not the major mechanism for cell death induced by the combination of Napa and Ref‐1 inhibitors observed in Figure 3.

### 3.5 PDAC tumours grown in the presence of CAFs are more sensitive to Ref‐1 inhibition in combination with STAT3 pathway inhibition via ruxolitinib

**Figure 5:**
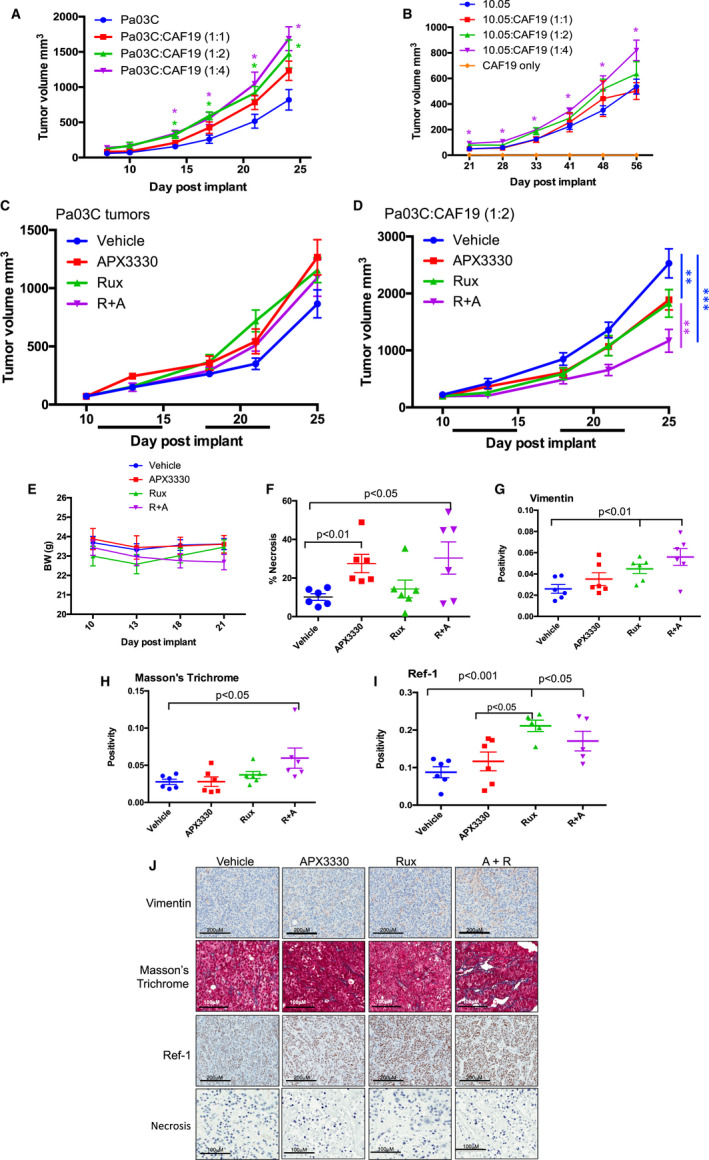
Dual targeting of Ref‐1 via APX3330 and STAT3 via ruxolitinib is effective in a tumour model with co‐implantation of CAFs. Tumour growth enhancement with the addition of CAFs to Pa03C cells (A) and Panc10.05 cells (B) over time. Varying tumour:CAF ratios were tested (n = 5‐7 mice, avg ± SE, *P < .05, **P < .01, ***P < .001). Pa03C tumours alone (C) or co‐implanted with CAFs at a 1:2 ratio (D) were treated with APX3330 (50 mg/kg, BID), Rux (50 mg/kg, SID), or APX3330+Rux (R+A; n = 7, **P < .01, ***P < .001, black lines indicate times of treatment). Bodyweights of the treated mice are shown in E. IHC of the tumours at sacrifice with H&E (F, *P* < .05, 0.01), vimentin (G, *P* < .01), Masson's trichrome (H, *P* < .05) and Ref‐1 (I, *P* < .05, 0.01) with representative pictures in J

To study the impact of CAF cells on tumour growth and response to combination therapy, mice were implanted with either tumour cells alone or co‐implanted with CAFs at varying ratios. After 24 days of growth, Pa03C cells with a ratio of either 1:2 or 1:4 CAF cells had significantly larger tumour volume than Pa03C cells alone (Figure 5A). The response in Panc10.05 was similar, yet not as robust, with significantly larger tumours in the ratio of 1:4 (Figure 5B). If CAFs were implanted alone, there was no tumour formation (Figure 5B). As expected, the addition of CAFs stimulates the growth of pancreatic cancer cells and accelerates the tumour growth rate significantly.

In a second study of CAF cells in response to combination therapy, Pa03C cells were implanted into mice, either alone or in conjunction with CAF19 cells (1:2 ratio) and allowed to grow for 11 days. Mice were then given APX3330 (50 mg/kg), Rux (50 mg/kg), or a combination of both via gavage five days a week for two weeks. At these doses there was no difference in tumour volume in mice implanted with tumour cells alone (Figure 5C). However, tumours co‐implanted with CAFs demonstrated growth inhibition under all conditions. We observed a ~25% decrease in tumour volume with single agents compared to vehicle control, and the combination of APX3330 and Rux resulted in the greatest response, about 50% smaller than vehicle (Figure 5D, **P < .01, ***P < .001). We also estimated the growth rate per day of the tumour volume among the groups using repeated measure regression model and determined that all the growth rates of the tumours in the graph in Figure 5D were significantly different (*P* < .01) except for APX3330 and Rux treatment as single agents. Combination treatment was well tolerated as demonstrated by no significant differences in bodyweights (Figure 5E). Immunohistochemistry including H&E, Masson's trichrome, Vimentin and Ref‐1 was performed on the tumours that responded to the APX‐Rux treatment (Figure 5F‐J). An increase in tumour necrosis of about 15% over vehicle control was observed with APX3330 treatment (*P* < .01) or APX3330 and Rux (*P* < .05, Figure 5F,J). Tumours also demonstrated increased positivity for the CAF marker, vimentin (*P* < .01) in Rux as a single agent and in combination treatment over vehicle (Figure 5G,J). This indicates that CAF cells were not killed by treatment, and these results were confirmed with greater positivity staining with Masson's trichrome (*P* < .05, Figure 5H,J) in combination treatment. Similarly, Ref‐1 staining was increased in tumours from mice that received Rux as a single agent and combination APX + Rux treatment compared to vehicle (*P* < .05, Figure 5I,J). Overall, PDAC cells in the presence of CAF cells were more sensitive to STAT3 and Ref‐1 inhibition, with the greatest tumour reduction present in combination treatment, and no reduction in the CAFs as determined by vimentin and Masson's trichrome.

### 3.6 Inhibition of STAT3 via napabucasin in combination with Ref‐1 inhibition is more effective at preventing tumour growth when grown in the presence of CAFs

**Figure 6:**
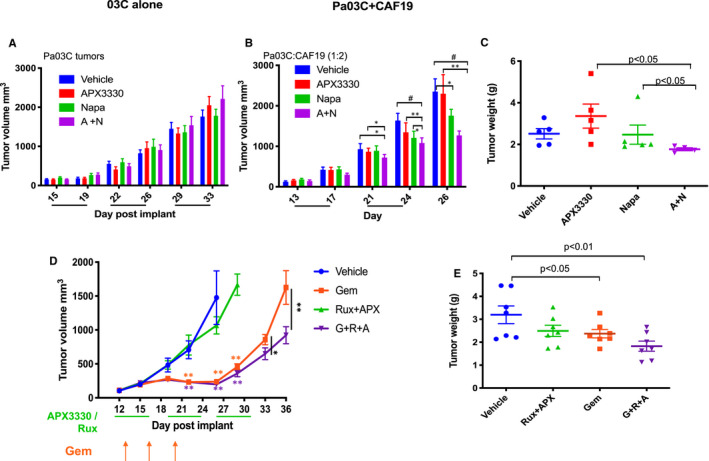
Combination of napabucasin and APX3330 is only effective when the CAFs are present and enhancement of standard of care agent, gemcitabine can be achieved using a tumour model with co‐implantation of CAFs. Pa03C tumours alone (A) or co‐implanted with CAFs at a 1:2 ratio (B) were treated with APX3330 (25 mg/kg, BID), Napa (50 mg/kg, BID), or APX3330+Napa (A+N; n = 5‐6, *P < .05, **P < .01, ***P < .001, black lines indicate times of treatment), with tumor weights in C that correspond to tumor volumes in B. Standard of care agent, gemcitabine was also added to the in vivo regimen (orange arrows) and the three‐drug combination grew significantly slower than the other groups (D, *P < .05, ** *P* < .01, green lines indicate times of treatment, orange and purple stars are compared to vehicle). Pa03C tumours co‐implanted with CAFs were treated with APX3330 (50 mg/kg, BID, PO)+Rux (50 mg/kg, SID, PO), Gemcitabine alone (35 mg/kg, days 13, 16, 20, ip) or APX3330+Rux+Gemcitabine (G+R+A; n = 7, *P < .05, **P < .01) with tumour weights in E (*P < .05, **P < .01). Orange and purple stars (*) are compared to vehicle control and black star (*) compared to Gem alone

As a further confirmation of the effects of dual inhibition of Ref‐1 and STAT3 in PDAC tumours co‐implanted with CAFs, we performed an in vivo study using the stem cell/ STAT3 inhibitor Napa. Mice were implanted with Pa03C alone or Pa03C and CAF19 cells. Tumours were allowed to grow to ~150 mm^3^ and then treatment with APX3330 (25 mg/kg) and Napa (50 mg/kg) began twice daily. Similarly, to the tumour growth in Figure 5C, when Pa03C cells were implanted alone, there was no significant difference between tumour growth regardless of treatment conditions (Figure 6A). However, in tumours co‐implanted with CAFs, treatment with the combination of APX3330 and Napa resulted in tumour volumes ~46% smaller than vehicle control (*P* < .02, Figure 6B). Napa treatment alone decreased the tumour volume by ~25% (*P* < .05, Figure 6B). APX3330 at the 25 mg/kg dose (Figure 6C) was not effective in reducing tumour volume compared to the 50 mg/kg dose shown in Figure 5D. The effects of the combination treatment on tumour volume are consistent with those from the Rux treated mice, confirming that overall, a combination STAT3 and Ref‐1 inhibitors reduced tumour growth in the presence of the appropriate microenvironment.

In a more aggressive treatment regimen that included standard of care agent gemcitabine, mice were challenged with gemcitabine alone or gemcitabine in combination with Ref‐1 and STAT3 inhibition. Mice were co‐implanted with Pa03C and CAF19 cells at 1:2 ratio and then treated with gemcitabine (35 mg/kg), APX3330+Rux, or gemcitabine+APX3330+Rux (Figure 6D). Again, we estimated the growth rate per day of the tumour volume among the groups using repeated measure regression model and determined that all the slopes of the lines in the graph in Figure 6D were significantly different (*P* < .01). The slope difference between the gemcitabine alone and the gemcitabine+APX3330+Rux regimen was also significantly different (*P* < .01). Mice were killed for tumour weight measurements when tumours reached 2000 mm^3^ or after 36 weeks. Tumours were collected and weighed, and in mice treated with gemcitabine+APX3330+Rux regimen or gemcitabine alone, the tumours were significantly smaller than vehicle tumours (*P* < .01). This tumour size reduction is significant considering that the treated tumours were allowed to grow an additional 13 days past vehicle‐treated mice (Figure 6E). The APX+Rux combination was allowed to grow for an additional 4 days and was nearing significance (*P* = .07, Figure 6E). These results further implicate that STAT3 and Ref‐1 inhibition are effective in PDAC tumour reduction and may enhance the response to standard of care agent, gemcitabine.

## 4 DISCUSSION

Although there has been little progress in the long‐term survival rates of patients with PDAC, in the metastatic setting combinations of gemcitabine/Abraxane (nab‐paclitaxel) or FOLFIRINOX (5‐FU/irinotecan/leucovorin/oxaliplatin) have increased response rates and median overall survival by several months.^55^, ^56^, ^57^ There is clearly a need for the development of new, multi‐targeted combinations that can positively impact the progression of this disease. Our data suggest that inhibiting the Ref‐1–STAT3 axis leads to a transcriptional reprogramming in tumour and/or microenvironment through alterations in effector proteins and will make an impact on the pathways regulated by these proteins, for example proliferation, invasion and response to hypoxic conditions.^3^, ^58^ In the studies presented here, Gemcitabine was added to the APX+Rux regimen due to its widespread use in PDAC as well as several previously published studies demonstrating the increased efficacy of gemcitabine when used in combination with perturbation of STAT3 signalling.^59^, ^60^, ^61^, ^62^, ^63^ We demonstrated that the addition of Gemcitabine to dual targeting of Ref‐1 and STAT3 was more efficacious than either combination alone.

Both Ref‐1 and STAT3 regulate downstream targets that play a role in tumour and stromal cell proliferation, response to hypoxia, and cytokine production.^14^, ^59^, ^64^, ^65^, ^66^, ^67^ Our previous work demonstrated that relevant stimuli found within the PDAC microenvironment, IL‐6 and hypoxia definitely stimulate interactions between Ref‐1 and one of its redox targets, STAT3.^43^ In general, STAT3 signalling promotes a tumour microenvironment that enables the tumour to proliferate and spread as well as avoid the immune system; within the tumour, STAT3 regulates pathways involved in cell proliferation, viability, angiogenesis and metastases.^59^, ^68^, ^69^, ^70^, ^71^, ^72^, ^73^ Inhibition of STAT3 signalling decreases the expression of genes that provide the tumour a growth advantage as well as suppressing the effects coming from cells in the microenvironment.^48^, ^66^, ^69^, ^74^, ^75^ 3D co‐culture models as well as PDAC xenolines co‐implanted with CAFs were used to determine the effects on tumour growth in 3D and in vivo following treatment with Ref‐1 and STAT3 inhibitors.

Potent, selective STAT3 inhibitors have been somewhat elusive to develop as many groups over time have attempted to target STAT3 both as an important target within the tumour itself as well as the role it plays in the tumour microenvironment.^13^, ^48^ In this work, we chose two inhibitors that have been used successfully in other clinical settings: Rux and Napa. We used Rux in these studies to inhibit STAT3 signalling through JAK1/2, and we expanded upon our previous study and further demonstrated a lack of phosphorylation after Rux treatment in treated PDAC cells.^3^ Due to our published data demonstrating the synergistic effects of targeting the Ref‐1–STAT3 axis, we anticipated that APX3330 exposure would augment Rux‐mediated inhibition especially in models of pancreatic cancer that express high levels of Ref‐1 and STAT3. Although Rux in combination with capecitabine (a prodrug of 5‐FU) did not demonstrate superior activity in PDAC patients, it is possible that Rux could be utilized in a combination regimen that would lead to improved patient response in other rationally selected inhibitor combinations.^76^, ^77^ For example, Gore et al present data to suggest that there is a subset of PDAC patients that have an angiogenic phenotype that would respond to Rux and in a mouse model of PDAC (KRC) Rux could extend survival.^74^ One explanation for the failure of enhanced efficacy in the Rux+Capecitabine clinical trial could centre on the lack of focusing on enhancing the inhibition of the JAK/STAT pathway at multiple points along that axis. The other STAT3 inhibitor that we used was Napa (or BBI‐608). Napa was shown to inhibit STAT3 transcription leading to a decrease in stem‐like properties of pancreatic and colon cancer cells.^18^ It also inhibits p‐STAT3 levels following treatment in PDAC cells (Figure 2) as well as inhibition of direct STAT3 targets including survivin, c‐Myc and Nanog in prostate cancer cells.^16^ It can also be bioactivated by NQO1 resulting in ROS production in pancreatic cancer cell lines.^17^ Phase III data in colon cancer patients demonstrate that in patients with high levels of p‐STAT3 overall survival was greater in Napa‐treated patients.^15^ Napa is currently being investigated in the clinic for multiple cancer indications where STAT3 signalling is believed to be important, which could result in rapid translation in PDAC. We observe dramatic, significant inhibition of 3D co‐culture tumour growth upon combination treatment with APX compounds and Napa (Figure 2). This effect is specific to the cancer cells as the CAFs in the 3D co‐culture did not seem to be overly affected by the combination and this could be due to the increase in cancer stemness of the tumour cells in comparison to the CAFs.^18^ This is important to note and a strength of our 3D co‐culture assay as more studies continue to demonstrate that depletion of the stroma does not decrease the progression of the disease.^78^ Our efforts are aimed at discovering a therapeutic combination that is efficacious at killing the tumour in the presence of its protective stroma. However, there is still much to discover regarding the role of STAT3 signalling in CAFs as well as Napa's mechanism of action (as evidenced by the extreme sensitivity of cell lines that express NQO1), and we fully acknowledge that there could be additional mechanisms that lead to the dramatic cell killing that we observe with Napa treatment alone or in combination treatment of APX compounds and Napa. A large induction of ROS with the combination treatment was not observed, therefore we do not think ROS induction is the main mechanism of action. A dramatic burst of ROS was not observed with Napa treatment alone, however the caveat is that CellRox Green is most responsive to superoxide and hydroxl radicals than to other radicals. There could be additional forms of ROS that are generated with NQO1 activation of Napa. Our data in monolayer, 3D co‐culture, and in vivo all strongly point towards dual targeting of Ref‐1 and STAT3 to being deleterious to pancreatic cancer cells, when appropriate combinations are used.

Targeting multiple aspects of the tumour signalling network is key to improving success in treating pancreatic cancer – a disease with one of the worst 5‐year survival rates of any cancer. This is partially due to the genomic heterogeneity found in human PDAC samples.^79^, ^80^, ^81^, ^82^ Previous clinical and preclinical studies suggest that multi‐targeted combination treatments that synergize will be more efficacious. Consequently, we interrogated two proteins that can transcriptionally reprogram the pancreatic tumour cells affecting multiple pathways critical to tumour survival and cross talking with many of the resistance pathways. An increase in STAT3 signalling is likely to occur through extrinsic signals such as IL‐6 or hypoxia and Ref‐1 would play a role in fully activating HIF1‐α, NFκB, AP‐1 in addition to STAT3. Therefore, our approach was to target two critical proteins, Ref‐1 and STAT3, whereby the propagation of signals between tumour and its microenvironment can potentially be blocked leading to a sensitization of the tumour to chemotherapy leading to a tumour‐induced cell death. One very important takeaway from the work presented here is the clear role that the CAFs play in the tumours’ response to this targeted therapy. Without the CAFs present in the in vivo models, the impact on the dual targeting of Ref‐1 and STAT3 was not significant. However, upon the addition of the relevant microenvironment, the importance of the Ref‐1STAT3 axis became obvious and had a great impact on tumour growth. These studies underscore the importance of using clinically relevant models that consist of cells that are appropriate for the microenvironment of the tumour.

PDAC pathways are significantly changed when Ref‐1 expression is decreased including STAT3, hypoxia signalling (HIF1), and apoptosis, again strongly supporting Ref‐1 as a target in PDAC and interaction between the Ref‐1 and STAT3 signalling.^47^, ^83^ Importantly, although multiple pathways may be modulated, inhibition of Ref‐1 and STAT3 was well tolerated in studies presented here as well as previously published animal and human studies.^10^, ^84^, ^85^

For future directions, FOLFIRINOX will also be tested in combination with Ref‐1–STAT3 targeting. This drug combination is being increasingly used in the treatment of PDAC patients. Two recent publications demonstrate that manipulation of the JAK/STAT signalling in combination with oxaliplatin, a component of FOLFIRiNOX, is more efficacious and may play a role in the cells’ inherent resistance to targeted agents.^86^, ^87^ Furthermore, knocking down STAT3 in mutant KRAS colorectal cancer cells, but not wild‐type KRAS cells resulted in enhancement of cells’ response to oxaliplatin and 5‐FU.^87^ As KRAS is mutated in >95% of PDAC patients, we will interrogate how the Ref‐1‐STAT3 axis affects the cells' response to FOLFIRINOX. There are other worthwhile drug combinations to pursue based on the signalling pathways that the Ref‐1/STAT3 axis affect. One example is immunotherapy as Mace *et al* demonstrate that dual targeting of IL‐6 with anti‐PD‐L1 antibody is efficacious in orthotopic and subcutaneous mouse models.^88^ Evaluation of novel combination therapy is critically important for therapeutic options for PDAC patients remain severely limited. Our genetic and pharmacological studies also point towards an essential molecular interplay between Ref‐1 and STAT3 that controls survival in PDAC and yet largely leaves the CAF cells unaffected.^3^, ^6^, ^89^ This investigation into drug synthetic lethality provides rationale that through a more detailed understanding of the tumour–CAF crosstalk and signalling mechanisms we can devise strategies to kill pancreatic cancer cells even in the protective environment of the CAFs.^90^

## CONFLICTS OF INTEREST

Mark R. Kelley has licensed APX3330 through Indiana University Research and Technology Corporation to Apexian Pharmaceuticals LLC. APX2009 and APX2014 are second‐generation compounds from Apexian Pharmaceuticals. Apexian Pharmaceuticals had neither control nor oversight of the studies, interpretation, or presentation of the data in this manuscript. Apexian Pharmaceuticals has sublicensed the APX compounds to Ocuphire Pharma who also had no control nor oversight of studies, interpretation, or presentation of the data in the manuscript.

## AUTHOR CONTRIBUTIONS

**Rachel Caston**: Investigation (supporting); Writing‐original draft (equal); Writing – review and editing (equal). **Fenil Shah**: Conceptualization (supporting); Data curation (supporting); Investigation (equal); Writing – review and editing (supporting). **Colton Starcher**: Investigation (supporting); Writing – review and editing (supporting). **Randall Wireman**: Investigation (supporting); Methodology (supporting); Writing – review and editing (supporting). **Olivia Babb**: Data curation (supporting); Writing – review and editing (supporting). **Michelle Grimard**: Investigation (supporting). **Jack McGeown**: Investigation (supporting). **Lee Armstrong**: Investigation (supporting); Writing – review and editing (supporting). **Yan Tong**: Formal analysis (supporting); Investigation (supporting); Writing – review and editing (supporting). **Roberto Pili**: Resources (supporting). **Joseph E Rupert**: Investigation (supporting); Resources (supporting). **Teresa A. Zimmers**: Conceptualization (equal); Resources (supporting); Supervision (supporting); Writing – review and editing (supporting). **Adily Elmi**: Investigation (supporting). **Karen Pollok**: Resources (supporting); Writing – review and editing (supporting). **Edward Motea**: Supervision (supporting); Validation (supporting); Writing – review and editing (supporting). **Mark Kelley**: Conceptualization (equal); Funding acquisition (equal); Project administration (equal); Resources (lead); Supervision (equal); Writing – original draft (lead). **Melissa Fishel**: Conceptualization (lead); Funding acquisition (equal); Investigation (equal); Methodology (equal); Project administration (lead); Supervision (lead); Visualization (lead); Writing – original draft (lead); Writing – review and editing (equal).

## Supporting information

Supplementary Material

## Data Availability

There was no big data or datasets generated in this manuscript.
