# Supplementary material for: Combined inhibition of Ref‐1 and STAT3 leads to synergistic tumour inhibition in multiple cancers using 3D and in vivo tumour co‐culture models
Source: J Cell Mol Med. 2020 Dec 3;25(2):784–800. doi: 10.1111/jcmm.16132 (PMC7812272; doi:10.1111/jcmm.16132)
Supplement: Supplementary file 1 — Supplementary Material [file JCMM-25-784-s001.pptx]

## Slide 1
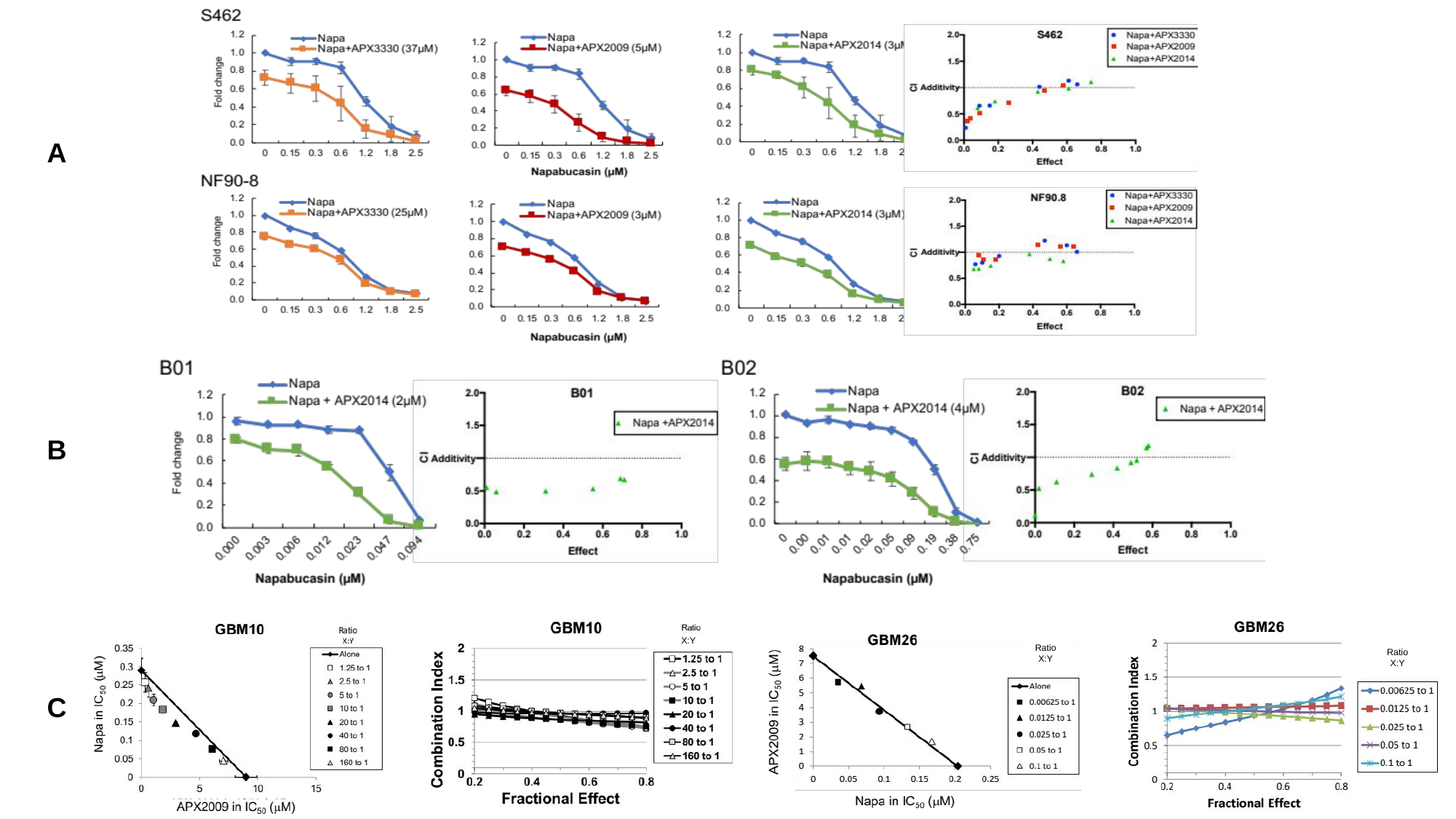

A
B
X:Y
Ratio
X:Y
Ratio
X:Y
C

## Slide 2
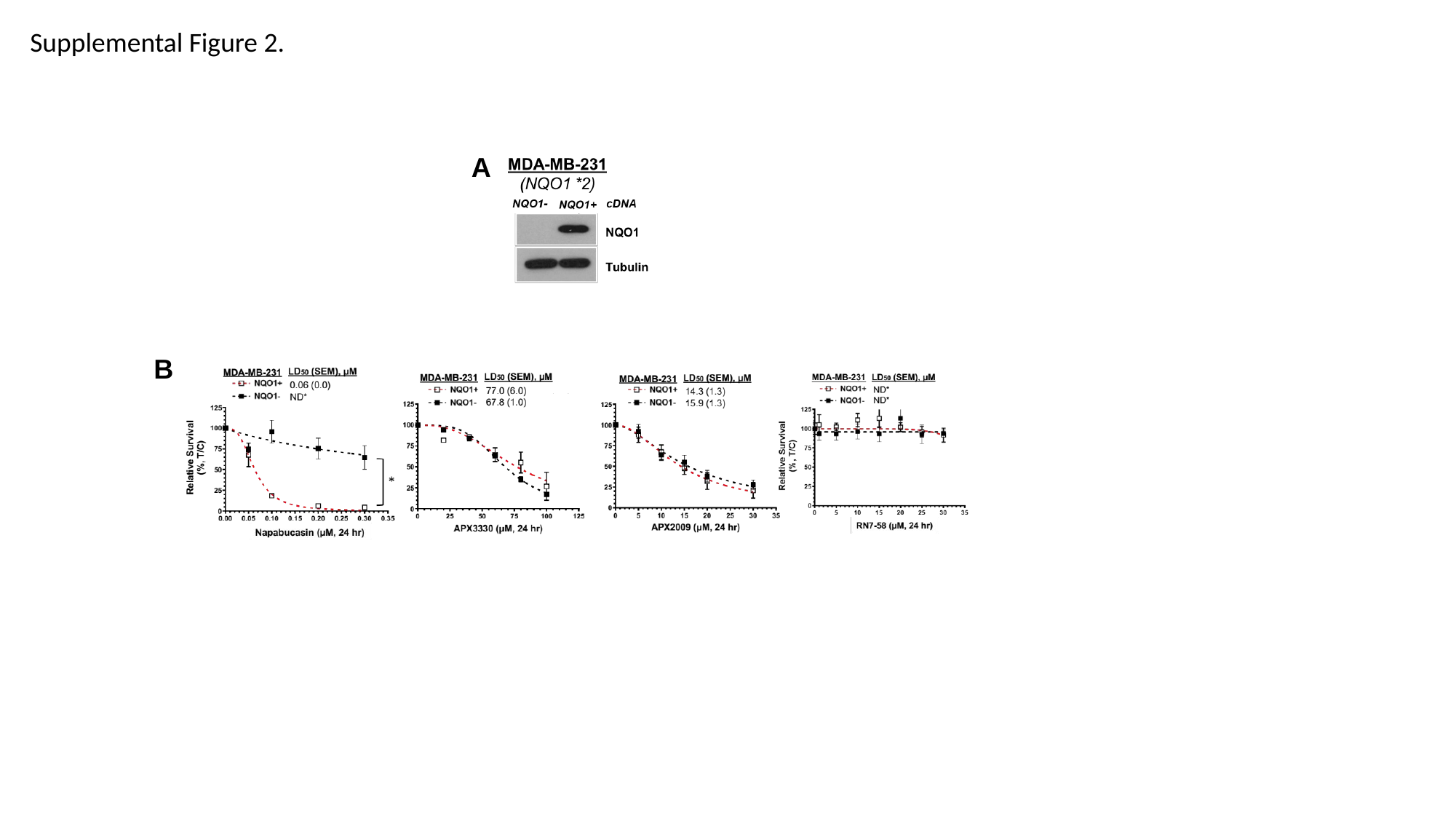

Supplemental Figure 2.
A
B

## Slide 3
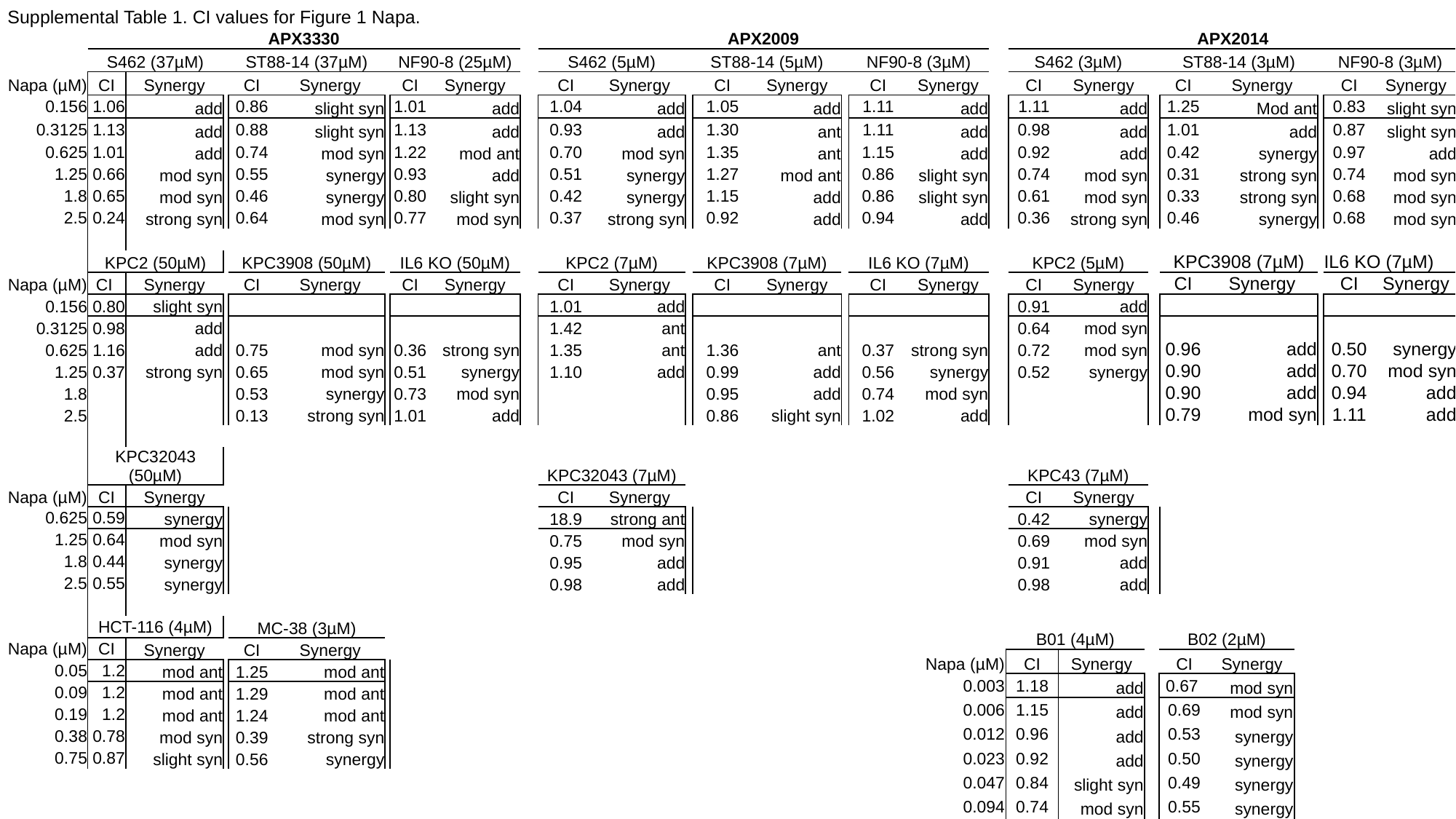

Supplemental Table 1. CI values for Figure 1 Napa.
| | APX3330 | | | | | | | | | APX2009 | | | | | | | | | APX2014 | | | | | | | |
| --- | --- | --- | --- | --- | --- | --- | --- | --- | --- | --- | --- | --- | --- | --- | --- | --- | --- | --- | --- | --- | --- | --- | --- | --- | --- | --- |
| | S462 (37µM) | | | ST88-14 (37µM) | | | NF90-8 (25µM) | | | S462 (5µM) | | | ST88-14 (5µM) | | | NF90-8 (3µM) | | | S462 (3µM) | | | ST88-14 (3µM) | | | NF90-8 (3µM) | |
| Napa (µM) | CI | Synergy | | CI | Synergy | | CI | Synergy | | CI | Synergy | | CI | Synergy | | CI | Synergy | | CI | Synergy | | CI | Synergy | | CI | Synergy |
| 0.156 | 1.06 | add | | 0.86 | slight syn | | 1.01 | add | | 1.04 | add | | 1.05 | add | | 1.11 | add | | 1.11 | add | | 1.25 | Mod ant | | 0.83 | slight syn |
| 0.3125 | 1.13 | add | | 0.88 | slight syn | | 1.13 | add | | 0.93 | add | | 1.30 | ant | | 1.11 | add | | 0.98 | add | | 1.01 | add | | 0.87 | slight syn |
| 0.625 | 1.01 | add | | 0.74 | mod syn | | 1.22 | mod ant | | 0.70 | mod syn | | 1.35 | ant | | 1.15 | add | | 0.92 | add | | 0.42 | synergy | | 0.97 | add |
| 1.25 | 0.66 | mod syn | | 0.55 | synergy | | 0.93 | add | | 0.51 | synergy | | 1.27 | mod ant | | 0.86 | slight syn | | 0.74 | mod syn | | 0.31 | strong syn | | 0.74 | mod syn |
| 1.8 | 0.65 | mod syn | | 0.46 | synergy | | 0.80 | slight syn | | 0.42 | synergy | | 1.15 | add | | 0.86 | slight syn | | 0.61 | mod syn | | 0.33 | strong syn | | 0.68 | mod syn |
| 2.5 | 0.24 | strong syn | | 0.64 | mod syn | | 0.77 | mod syn | | 0.37 | strong syn | | 0.92 | add | | 0.94 | add | | 0.36 | strong syn | | 0.46 | synergy | | 0.68 | mod syn |
| | | | | | | | | | | | | | | | | | | | | | | | | | | |
| | KPC2 (50µM) | | | KPC3908 (50µM) | | | IL6 KO (50µM) | | | KPC2 (7µM) | | | KPC3908 (7µM) | | | IL6 KO (7µM) | | | KPC2 (5µM) | | | KPC3908 (7µM) | | | IL6 KO (7µM) | |
| Napa (µM) | CI | Synergy | | CI | Synergy | | CI | Synergy | | CI | Synergy | | CI | Synergy | | CI | Synergy | | CI | Synergy | | CI | Synergy | | CI | Synergy |
| 0.156 | 0.80 | slight syn | | | | | | | | 1.01 | add | | | | | | | | 0.91 | add | | | | | | |
| 0.3125 | 0.98 | add | | | | | | | | 1.42 | ant | | | | | | | | 0.64 | mod syn | | | | | | |
| 0.625 | 1.16 | add | | 0.75 | mod syn | | 0.36 | strong syn | | 1.35 | ant | | 1.36 | ant | | 0.37 | strong syn | | 0.72 | mod syn | | 0.96 | add | | 0.50 | synergy |
| 1.25 | 0.37 | strong syn | | 0.65 | mod syn | | 0.51 | synergy | | 1.10 | add | | 0.99 | add | | 0.56 | synergy | | 0.52 | synergy | | 0.90 | add | | 0.70 | mod syn |
| 1.8 | | | | 0.53 | synergy | | 0.73 | mod syn | | | | | 0.95 | add | | 0.74 | mod syn | | | | | 0.90 | add | | 0.94 | add |
| 2.5 | | | | 0.13 | strong syn | | 1.01 | add | | | | | 0.86 | slight syn | | 1.02 | add | | | | | 0.79 | mod syn | | 1.11 | add |
| | | | | | | | | | | | | | | | | | | | | | | | | | | |
| | KPC32043 (50µM) | | | | | | | | | KPC32043 (7µM) | | | | | | | | | KPC43 (7µM) | | | | | | | |
| Napa (µM) | CI | Synergy | | | | | | | | CI | Synergy | | | | | | | | CI | Synergy | | | | | | |
| 0.625 | 0.59 | synergy | | | | | | | | 18.9 | strong ant | | | | | | | | 0.42 | synergy | | | | | | |
| 1.25 | 0.64 | mod syn | | | | | | | | 0.75 | mod syn | | | | | | | | 0.69 | mod syn | | | | | | |
| 1.8 | 0.44 | synergy | | | | | | | | 0.95 | add | | | | | | | | 0.91 | add | | | | | | |
| 2.5 | 0.55 | synergy | | | | | | | | 0.98 | add | | | | | | | | 0.98 | add | | | | | | |
| | | | | | | | | | | | | | | | | | | | | | | | | | | |
| | HCT-116 (4µM) | | | MC-38 (3µM) | | | | | | | | | | | | | | | | | | | | | | |
| Napa (µM) | CI | Synergy | | CI | Synergy | | | | | | | | | | | | | | | | | | | | | |
| 0.05 | 1.2 | mod ant | | 1.25 | mod ant | | | | | | | | | | | | | | | | | | | | | |
| 0.09 | 1.2 | mod ant | | 1.29 | mod ant | | | | | | | | | | | | | | | | | | | | | |
| 0.19 | 1.2 | mod ant | | 1.24 | mod ant | | | | | | | | | | | | | | | | | | | | | |
| 0.38 | 0.78 | mod syn | | 0.39 | strong syn | | | | | | | | | | | | | | | | | | | | | |
| 0.75 | 0.87 | slight syn | | 0.56 | synergy | | | | | | | | | | | | | | | | | | | | | |
| | B01 (4µM) | | | B02 (2µM) | | |
| --- | --- | --- | --- | --- | --- | --- |
| Napa (µM) | CI | Synergy | | CI | Synergy | |
| 0.003 | 1.18 | add | | 0.67 | mod syn | |
| 0.006 | 1.15 | add | | 0.69 | mod syn | |
| 0.012 | 0.96 | add | | 0.53 | synergy | |
| 0.023 | 0.92 | add | | 0.50 | synergy | |
| 0.047 | 0.84 | slight syn | | 0.49 | synergy | |
| 0.094 | 0.74 | mod syn | | 0.55 | synergy | |

## Slide 4
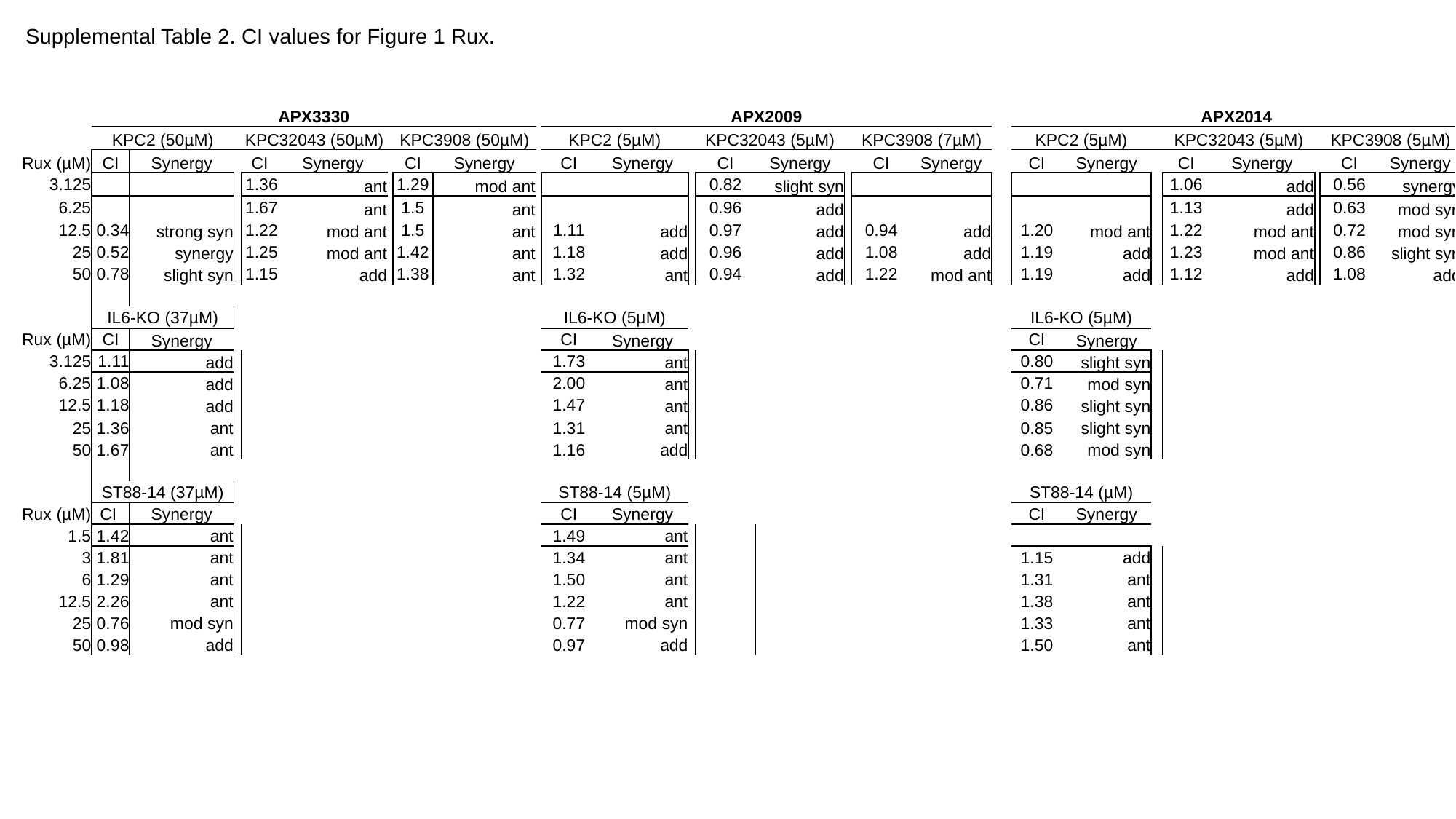

Supplemental Table 2. CI values for Figure 1 Rux.
| | APX3330 | | | | | | | | | APX2009 | | | | | | | | | APX2014 | | | | | | | |
| --- | --- | --- | --- | --- | --- | --- | --- | --- | --- | --- | --- | --- | --- | --- | --- | --- | --- | --- | --- | --- | --- | --- | --- | --- | --- | --- |
| | KPC2 (50µM) | | | KPC32043 (50µM) | | | KPC3908 (50µM) | | | KPC2 (5µM) | | | KPC32043 (5µM) | | | KPC3908 (7µM) | | | KPC2 (5µM) | | | KPC32043 (5µM) | | | KPC3908 (5µM) | |
| Rux (µM) | CI | Synergy | | CI | Synergy | | CI | Synergy | | CI | Synergy | | CI | Synergy | | CI | Synergy | | CI | Synergy | | CI | Synergy | | CI | Synergy |
| 3.125 | | | | 1.36 | ant | | 1.29 | mod ant | | | | | 0.82 | slight syn | | | | | | | | 1.06 | add | | 0.56 | synergy |
| 6.25 | | | | 1.67 | ant | | 1.5 | ant | | | | | 0.96 | add | | | | | | | | 1.13 | add | | 0.63 | mod syn |
| 12.5 | 0.34 | strong syn | | 1.22 | mod ant | | 1.5 | ant | | 1.11 | add | | 0.97 | add | | 0.94 | add | | 1.20 | mod ant | | 1.22 | mod ant | | 0.72 | mod syn |
| 25 | 0.52 | synergy | | 1.25 | mod ant | | 1.42 | ant | | 1.18 | add | | 0.96 | add | | 1.08 | add | | 1.19 | add | | 1.23 | mod ant | | 0.86 | slight syn |
| 50 | 0.78 | slight syn | | 1.15 | add | | 1.38 | ant | | 1.32 | ant | | 0.94 | add | | 1.22 | mod ant | | 1.19 | add | | 1.12 | add | | 1.08 | add |
| | | | | | | | | | | | | | | | | | | | | | | | | | | |
| | IL6-KO (37µM) | | | | | | | | | IL6-KO (5µM) | | | | | | | | | IL6-KO (5µM) | | | | | | | |
| Rux (µM) | CI | Synergy | | | | | | | | CI | Synergy | | | | | | | | CI | Synergy | | | | | | |
| 3.125 | 1.11 | add | | | | | | | | 1.73 | ant | | | | | | | | 0.80 | slight syn | | | | | | |
| 6.25 | 1.08 | add | | | | | | | | 2.00 | ant | | | | | | | | 0.71 | mod syn | | | | | | |
| 12.5 | 1.18 | add | | | | | | | | 1.47 | ant | | | | | | | | 0.86 | slight syn | | | | | | |
| 25 | 1.36 | ant | | | | | | | | 1.31 | ant | | | | | | | | 0.85 | slight syn | | | | | | |
| 50 | 1.67 | ant | | | | | | | | 1.16 | add | | | | | | | | 0.68 | mod syn | | | | | | |
| | | | | | | | | | | | | | | | | | | | | | | | | | | |
| | ST88-14 (37µM) | | | | | | | | | ST88-14 (5µM) | | | | | | | | | ST88-14 (µM) | | | | | | | |
| Rux (µM) | CI | Synergy | | | | | | | | CI | Synergy | | | | | | | | CI | Synergy | | | | | | |
| 1.5 | 1.42 | ant | | | | | | | | 1.49 | ant | | | | | | | | | | | | | | | |
| 3 | 1.81 | ant | | | | | | | | 1.34 | ant | | | | | | | | 1.15 | add | | | | | | |
| 6 | 1.29 | ant | | | | | | | | 1.50 | ant | | | | | | | | 1.31 | ant | | | | | | |
| 12.5 | 2.26 | ant | | | | | | | | 1.22 | ant | | | | | | | | 1.38 | ant | | | | | | |
| 25 | 0.76 | mod syn | | | | | | | | 0.77 | mod syn | | | | | | | | 1.33 | ant | | | | | | |
| 50 | 0.98 | add | | | | | | | | 0.97 | add | | | | | | | | 1.50 | ant | | | | | | |
| | | | | | | | | | | | | | | | | | | | | | | | | | | |
| CI > 1.3 indicates antagonism, |
| --- |
| CI = 1.1 to 1.3 moderate antagonism, |
| CI = 0.9 to 1.1 additive effect, |
| CI = 0.8 to 0.9 slight synergism, |
| CI = 0.6 to 0.8 moderate synergism, |
| CI = 0.4 to 0.6 synergism, |
| CI = 0.2 to 0.4 strong synergism. |
